# Human gastrointestinal (GI) tract microbiome-derived pro-inflammatory neurotoxins from *Bacteroides fragilis*: Effects of low fiber diets and environmental and lifestyle factors

**Published:** 2020-03-09

**Authors:** Walter J Lukiw

**Affiliations:** 1 LSU Neuroscience Center, Louisiana State University Health Sciences Center, New Orleans LA 70112 USA; 2 Department of Ophthalmology, Louisiana State University Health Sciences Center, New Orleans LA 70112 USA; 3 Department of Neurology, Louisiana State University Health Sciences Center, New Orleans LA 70112 USA

## Overview

*Homo sapiens* harbor a complex and dynamic community of microorganisms, collectively known as ‘*the microbiome*’, that together constitute the largest ‘*dispersed organ system*’ on and within the body, cumulatively more massive, more metabolically active, and much more genetically complex than all of the multiple cell types of the human liver. Together with host cells and their genes, the microbiome constitutes the ‘*metaorganism*’, defined as an assemblage of interacting biological entities with significant commensal or symbiotic benefit to the entire lifeform. The human GI-tract microbiome’s dynamic complexity of different microbial species is largely dependent on diet, dietary fiber, environmental and lifestyle-factors, and the most recent evidence suggests that this in turn contributes to human behavior and immunological and neurological health and disease. This communication-perspectives article will briefly discuss the relatively recent research advances at the intersection of human GI-tract microbiome-derived pro-inflammatory neurotoxins and the effects of low-fiber diets, and environmental and lifestyle factors on microbial abundance and speciation. In addition to the nutrients obtained from our diet it is becoming increasingly clear that beneficial dietary effects on the maintenance of a healthy GI-tract microbiome may also reduce the abundance of pro-inflammatory neurotoxins with gastric, immunological and neurological implications. This paper will further focus on current research developments: **(i)** of one of the human GI-tract’s most abundant Gram-negative bacterial species *Bacteroides fragilis* (of the phylum *Bacteriodetes*); **(ii)** discuss recent advances in our understanding of the contribution of *B. fragilis*-derived pro-inflammatory neurotoxins and their noteworthy contribution to biophysical barrier disruption and systemic effects; and **(iii)** evaluate their potential influences on progressive, age-related inflammatory neurodegenerative disease such as those associated with the Alzheimer’s disease (AD) process.

## The human GI-tract microbiome

The microbiome of the human gastrointestinal (GI) tract contains by far the largest reservoir of microbes in the body, and is composed of about ~10^15^ microorganisms from many thousands of different microbial species; the latest estimate is that the number of microbial genes in the human GI-tract microbiome outnumber ‘*human* ‘*host*’ genes by about ~837 to 1 [1–9]. This tremendous genetic abundance and diversity of the cumulative microbiome genome and the transcription and translation of components needed for highly integrated biochemical signaling pathways forms the basis for GI-tract microbial influence on health and disease [9–15]. Especially over the last decade have we just begun to appreciate that the human GI-tract is a very bioactive and dynamic source of microorganisms that possess a staggering complexity and diversity [7–11]. The vast majority of the human GI-tract microbiota is composed of anaerobic or facultative anaerobic bacteria, with fungi, protozoa, Archaebacteria (an ancient intermediate microbial group between the prokaryotes and eukaryotes), viruses and other microorganisms making up the remainder. Interestingly, of all mammals so far characterized, human GI-tract microbial densities of up to 10^12^ per cm^**3**^ represent the highest recorded packing density of any known microbial ecosystem, and only 2 of the 52 known major bacterial divisions, currently identified by 16S rRNA sequencing and metagenomics analysis, are abundant in the average, healthy human GI-tract microbiome. These 2 major bacterial divisions include the Gram-positive *Firmicutes* (~51% of the total) and the anaerobic Gram-negative *Bacteroidetes* (~48%). The remaining 1% of microbiome-resident phylotypes are distributed amongst the *Cyanobacteria*, *Fusobacteria*, *Proteobacteria*, *Spirochetes* and *Verrucomicrobia*. In addition to various different species of Archaebacteria, fungi, protozoa, viruses and other commensal microorganisms these microorganisms make up the essential ‘*core*’ of the human GI-tract microbiome [4–9,16–22]. That the *Firmicutes* and *Bacteroidetes* were preferentially selected from the ~52 bacterial phyla available in the earth’s biosphere is of considerable evolutionary interest with implications for the ‘*hologenome*’ theory. This theory postulates that it is not the individual organism, but rather the host organism together with its associated symbiotic microbial communities, or complete ‘*metaorganism*’ with the repertoire of all genes that form the *‘metagenome*’ that should be considered as the basic unit of evolution, natural selection, genetic composition and potential for that individual organism [23–26]. Indeed the ‘*hologenome*’ theory incorporates the idea that the host along with its intracellular and extracellular microbiome functions as a unique and distinctive biological entity. Currently one of the most thoroughly studied and anatomically, developmentally, evolutionarily, immunologically and metabolically characterized phylum in the entire human GI-tract microbiome consist of the Gram-negative anaerobic bacillus of the genus *Bacteriodetes* and the abundant species *Bacteroides fragilis* [26–35].

## *Bacteriodetes* and *Bacteroides fragilis*

About ~99.5% of all of the microbes in the human GI-tract microbiome consist of facultative and/or obligate anaerobic bacteria depending mostly on their position along the human digestive/intestinal systems that measures about ~3.5 cm in diameter and approximately ~7 m in length [1–6,21,36–39]. Perhaps not too surprisingly the microbes in the deeper, more anaerobic parts of the small intestine are the most enriched in anaerobic microbial subtypes suggesting a robust adaptation to the pH and the biophysical and microbial composition of that segment of the GI-tract environment [14,15,21–31]. Within these specific GI-tract regions the most abundant Gram-negative bacteria of the human GI-tract microbiome consist mostly of the phylum *Bacteriodetes*, with a major genus-species being represented by the obligate Gram-negative anaerobe non-spore forming bacillus *Bacteroides fragilis*. The genus *Bacteroidetes* and the species *Bacteroides fragilis*: **(i)** are amongst the most studied and genetically understood human GI-tract resident microorganism [3,27–29,32–35]; **(ii)** lie at the core of the human GI-tract microbiome in both American and European populations [3,16,17]; **(iii)** exhibit a surprising amount of intra-species genomic diversity and associated range and variety of potential biochemical functions [3,5,34,35]; and **(iv)** can generate some of the most potent pro-inflammatory and pathogenic neurotoxins of all lifeforms yet studied [10–13,21,27–29]. Further, in these deep GI-tract regions *B. fragilis*: **(i)** are present at about one-hundred times the abundance of Gram negative bacilli of the phylum *Proteobacteria* and the genus-species *Escherichia coli* [28,29]; **(ii)** colonize this section of the human GI-tract at densities of up to 8 × 10^**10**^ CFU/cm^**3**^; this being the highest density of any microbial colonization known in nature [21–31]; **(iii)** reside and proliferate exclusively in the GI-tract of mammals [27–35]; and **(iv)** normally constitute an abundant repository of commensal, symbiotic bacteria generally highly beneficial to human immune-, digestive-, nutritive- and neurological health[31,32].

The significant health benefits of *Bacteriodetes* and *Bacteroides fragilis* in particular to human health is due in part to the remarkable ability of *Bacteroides* species to metabolize dietary fiber into volatile short chain fatty acids (SCFAs; including acetate, butyrate, lactate, propionate, valerate and other lipid nutrients) and/or to biosynthesize complex sugars and polysaccharides to maintain overall glucose homeostasis in multiple biophysiological compartments of the host, such as the systemic circulation, intracellular compartments and cerebrospinal fluid (CSF) [20,35,40]. SCFAs: **(i)** ordinarily function in the development, homeostasis, and maintenance of the host immune, neuro-endrocrine and digestive systems; and **(ii)** play an important regulatory role in glucose homeostasis, lipid metabolism and anti-inflammatory signaling in endothelial cells of the lining of the GI-tract, sometimes known as the intestinal endothelium [27–29]. Besides being of immense benefit to human general health in the extraction of energy from the diet, absorption of nutrients and generation of vitamins (such as vitamin B12 and K), amino acids (such as lysine) and peptide sugars (such as peptidoglycans), the microbiome anchors a robust systemic immune-defense system against infective pathogens [10–12,15–17]. Interestingly bacteria and microorganisms that make up the smallest 1% of the human GI-tract microbiome have a disproportionately large impact on, and relevance to, human disease, and a major function of abundant species normally residing in the healthy GI-tract microbiome is to regulate the proliferation of any potentially pathogenic microbes and keep them under homeostatic control [40–42].

On the other hand some of the most potent pathogenic and pro-inflammatory neurotoxins known originate from enterotoxigenic strains of the GI-tract microbiome-abundant anaerobic bacillus *Bacteroides fragilis* [27–35]. These include a group of extremely pro-inflammatory glycolipids known as lipopolysaccharide (LPS), lipooligosaccharide (LOS; truncated versions of ‘regular-sized’ LPS molecules), barrier-disrupting, cell-cell adhesion proteins, including E-cadherin cleaving-and-destroying enterotoxins such as *Bacteroides fragilis toxin* (BFT) or *fragilysin*, a large family of species-specific bacterial amyloids and information-carrying small non-coding RNAs (sncRNAs) similar in size and ribonucleic acid structure and composition to microRNAs (miRNAs) of the human host [43,44]. Together these *B. fragilis* exudates are known to negatively affect the structure and function of biophysiological barriers, such as the gastrointestinal (GI) mucosa that forms the basis for the GI-tract barrier, and the blood-brain barrier (BBB), to disrupt normal barrier-based selection and exclusion properties, enabling these toxic exudates to enter the systemic circulation and pass through the BBB to elicit inflammatory neurodegeneration and induce neuronal dysfunction, atrophy and loss in the CNS [13–18]. Very recently it has been shown that BF-LPS is abundant in AD brain neocortex, and in later stages of the disease can encapsulate neuronal nuclei of the neocortical hexalayer into ‘LPS cage’ structures and in doing so impair the exit of neuron-specific messenger RNA (mRNA) transcripts such as synapsin (SYN) and the neurofilament light (NF-L) chain protein [44–46]. Down-regulation of SYN and NF-L gene expression is an important characteristic of AD neuropathology and AD amyloids (such as Aβ42 peptide) appear to facilitate LPS entry into human neurons [45–47]. Another very recent finding is that the environmentally abundant neurotoxin aluminum sulfate significantly induces the generation of LPS from certain species of the human GI-tract microbiome-resident genus *Bacteriodetes* [48,49].

## Neurotoxins derived from the human GI-tract microbiome - GI-tract exudates – BF-LPS and fragilysin

The multiple strains of *Bacteroides fragilis* (*B. fragilis*) in the human GI-tract consist of two predominant subspecies - distinguished in part by their gene makeup, their genetic encoding capabilities and potential to biosynthesize and secrete: **(i)** extremely pro-inflammatory lipopolysaccharides such as BF-LPS; and **(ii)** to produce a zinc-dependent metalloprotease enterotoxin (sometimes recognized as a *metalloproteotoxin or metalloprotease*), also known as *B. fragilis* toxin (BFT) or *fragilysin* (EC 3.4.24.74) [33]. Species of *Bacteroides* that do not secrete BF-LPS or BFT (*fragilysin*) are termed ‘nontoxigenic *B. fragilis’* while those that do secrete BF-LPS or BFT are called ‘*enterotoxigenic’* strains of *B. fragilis* [32,33]. The GI-tract microbiome in addition secretes copious quantities of bacterial amyloids and small non-coding RNAs (sncRNAs) of which virtually nothing is known, or if they act independently or together to induce neuro-inflammation and neuropathology [43–49]. Within the last several years it has been demonstrated that *enterotoxigenic* strains of *B. fragilis* (ETBF) proliferate rapidly in the mammalian GI-tract both in the absence of adequate dietary fiber and in the presence of high-fat cholesterol (HF-C) diets [35,50–52]. This remarkable species propagation of a GI-tract resident microbe based on dietary fiber intake appears to enhance the intestinal abundance of *B. fragilis* and hence the potential of this Gram-negative obligate anaerobe to secrete its formidable array neurotoxic exudates. As mentioned previously these primarily include **(i)** the lipoglycan lipopolysaccharide (LPS), a particularly potent, pro-inflammatory LPS glycolipid subtype (BF-LPS) [10–12]; and **(ii)** the hydrolytic, extracellular zinc metalloproteinase known as *Bacteroides fragilis* toxin (BFT; also known as *fragilysin*); either alone or together these are respectively amongst the most pro-inflammatory lipoglycans and enterotoxins known [53–55]. For example, as quantified by the ability to generate the pro-inflammatory transcription factor NF-kB (p50/p65) in human neuronal-glial (HNG) cells in primary co-culture, BF-LPS was found to be the most inflammation-supporting factor in a large analytical panel of cytokines and amyloids, either alone or in combination [43,44]. The other major *B*. *fragilis*-derived, secreted enterotoxin BFT (*fragilysin*) has long been known to hydrolyze extracellular matrix proteins, and disrupt tight junctions of intestinal cells while also degrading intracellular and cytoskeletal proteins such as actin, myosin and other filamentous proteins [55,56]. BFT also causes significant oxidative DNA damage, epithelial membrane barrier damage and activation of pathogenic STAT3/Th17 immune responses [34,57]. Importantly, both BF-LPS and BFT (*fragilysin)* can leak through the normally protective mucosal barriers of the GI-tract intestinal endothelium to bring about substantial inflammatory pathology in both the systemic circulation and after BBB transit into vulnerable CNS compartments, including the highly vascularized neocortical parenchyma of the brain [29,53,55,58,59]. Indeed, while *Bacteroides fragilis* is an anaerobic bacillus and part of the normal microbiota of the human colon and is generally commensal, this microbe can cause a ‘smoldering’ systemic infection if displaced into the bloodstream or surrounding tissue following disease, trauma or surgery [58,60–66]. It is important to note that BF-LPS and BFT together have been detected both in the general circulation in patients exhibiting systemic inflammation, in the brains of amyloid over-expressing transgenic Alzheimer’s disease (AD) murine models and in the blood serum and parenchyma of advanced AD patients [66–69].

When the highly toxic exudates of enterotoxigenic strains of *B. fragilis* escape the microbial-dense environment of the human GI-tract they can produce substantial systemic inflammatory pathology with significant mortality and morbidity. *B. fragilis* proliferation and excess is associated with the development of multiple pro-inflammatory bowel cancers, bacteremia, brain and intra-abdominal abscess, cellulitis, colitis, diabetic ulcer, diarrhea, necrotizing fasciitis, peritonitis, sepsis, septicemia, systemic infection and systemic inflammation, the development of neurological diseases involving progressive, age-related inflammatory neurodegeneration (such as AD), and those neurological disorders that display a significantly elevated incidence of atypical developmental programming against a background of aging (such as schizophrenia) [37,70–76]. Very recently LPS-induced systemic inflammation has been associated with synaptic loss and cognitive decline in multiple human neurological disorders and in transgenic murine models for AD, and a role for LPS-mediated microglial release of pro-inflammatory cytokines, such as interleukin IL-1β, is currently based on both *in vivo* and primary culture studies *in vitro* [53,73].

## Diet and dietary fiber

While it is generally appreciated that healthy diets are replete with vitamins, essential trace metals, amino acids, fatty acids, lipids and nutrients for human health benefit it is not as well acknowledged that these same wholesome diets are also packed with both soluble and insoluble forms of dietary fiber. Generally, the term fiber refers to all the parts of plant-derived foods that cannot be easily digested or absorbed by the body. Soluble fiber is hydrophilic, water-soluble and turns into a gel-like constituency during digestion; examples are cereals, legumes of the family *Fabaceae* (or *Leguminosae*), fruits and vegetables of the families *Solanacea (tomatoe)*, *Brassicas (cruciferous vegetables)* and others such as seaweeds from the *Plantae* kingdoms of *chlorophytes, rhodophytes, phaeophytes* and *cyanophtyes*. On the other hand insoluble fiber does not dissolve in water and is left relatively intact as food moves through the GI-tract; important examples of insoluble fiber includes *‘hard’* grains such as barley (*Hordeum vulgare*) and whole grain rice (*Oryza sativa*), cruciferous vegetables of the family *Brassicaceae* and dark leafy vegetables. Perhaps as interesting as the contribution of *Firmicutes* and *Baceriodetes* to a healthy human microbiome is the contribution of *Fabaceae* or *Leguminosae or Brassicas* or other fiber-laden dietary nutrition to microbiome health, and serves as another interesting example of *‘interkingdom communication’*, ‘*beneficial plant-microbe interactions’* and dynamic signaling amongst selective species which inhabit our biosphere [73,75,77].

The average American diet is woefully inadequate in both soluble and insoluble forms of dietary fiber. For example for optimum health both the United States Food and Drug Administration (FDA) and the American Heart Association (AHA) suggest that the total daily fiber intake should range between 21–25 gm per day (for women under 50) and between 30–38 gm per day (for men under 50); while the average American diet contains only a fraction of this, averaging only 12–15 grams of total fiber per day or only 35–65% of what is termed as an ‘*ideal fiber intake*’ [74–78]. For one of the few thorough studies conducted in mammals (*Sus scrofa domesticus*) by Heinritz *et al.* reported that low-fat/high-fiber (LF/HF) diets stimulated beneficial bacteria and SCFA production while a high fat/low fiber (HF/LF) diet fostered the proliferation of those bacterial groups, including *B. fragilis*, associated with a negative impact on health conditions - concluding that there are important relationships amongst dietary fiber and fat intake, nutrition, gut microbial composition and host health [5,51]. In a more recent study in *Homo sapiens* the consumption of almonds, a rich source of both soluble and insoluble fiber, unsaturated fats, and polyphenols, all nutrients that can favorably alter the composition of the gut microbiome, were found to decrease the abundance of the pathogenic bacterium *Bacteroides fragilis* by 48% (overall relative abundance, *p*<0.05) [50]. It appears from multiple studies and from the evidence available that an adequate intake of dietary fiber is associated with digestive health and reduced risk for heart disease, stroke, hypertension, certain gastrointestinal disorders, obesity, type 2 diabetes, certain cancers such as colorectal cancer as well as neurological diseases ranging from schizophrenia to AD [13,34,37,42,43,50,57,74].

Several excellent and extensive recent reviews on the ability of fiber-rich foods to suppress the incidence and mortality from ‘Western’ diseases, notably cancers of the colon, breast, liver, cardiovascular, infectious, and respiratory diseases, diabetes, obesity and neurological disease have recently appeared and will not be dealt with further here. Most of these reviews are based on the original ideas of the British surgeon Denis Burkitt (1911–1993) and others on the ‘*dietary fiber hypothesis*’ and its important role in the maintenance of human health [75–79].

## Environment and lifestyle

**Figure 1.**
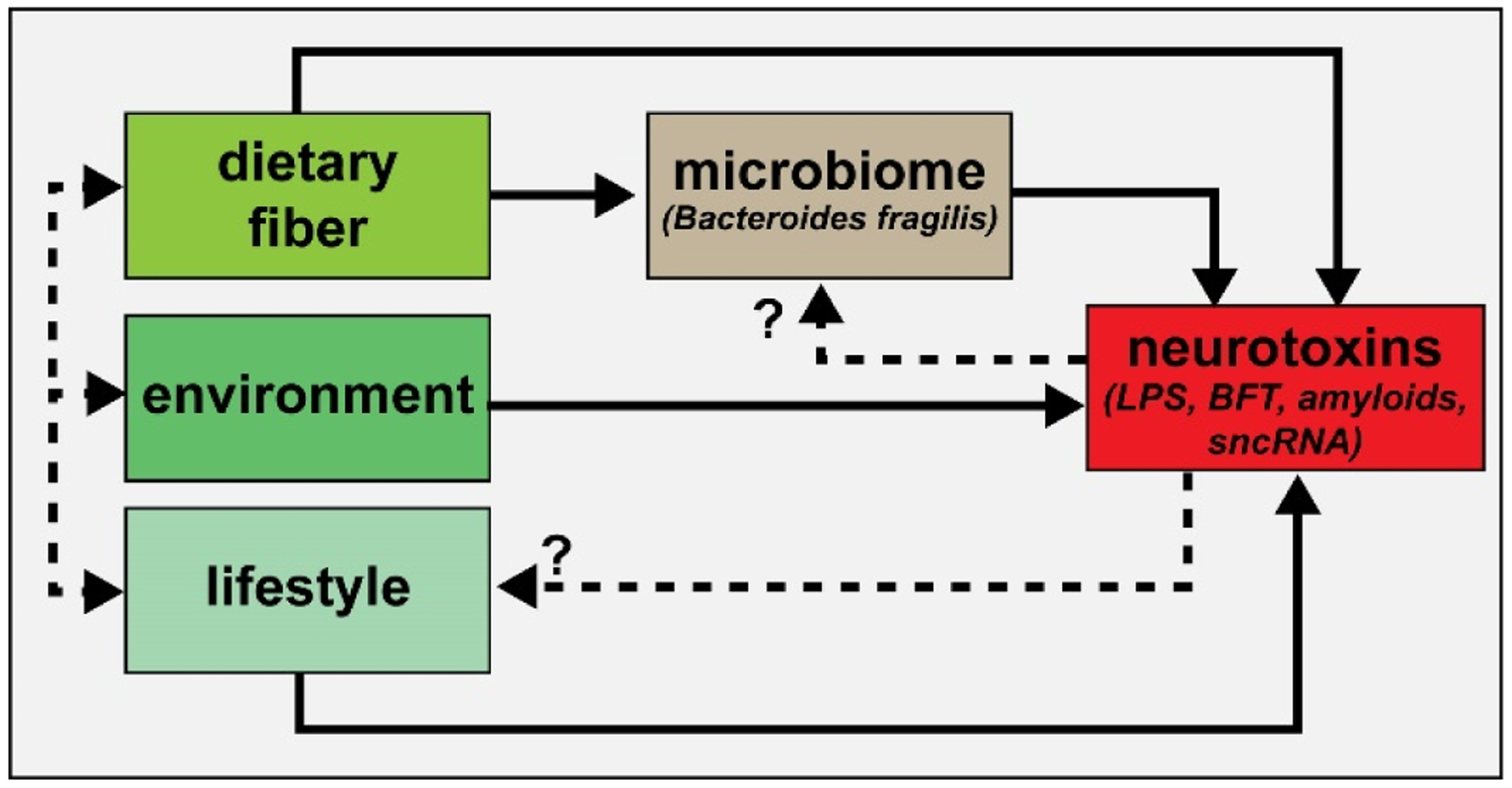
Highly simplified schematic of an integrated signaling system consisting of dietary-, environmental- and lifestyle-derived elements which provide neurotoxins – both neuro-inflammatory and neuro-pathogenic - to the brain and central nervous system (CNS). Dietary factors, such as both soluble and insoluble fiber from the diet are becoming increasingly appreciated as critical regulators of the abundance, speciation and health of microbial species in the microbiome [95–100]. In turn, abundant microbiome-resident Gram negative bacilli such as *Bacteroides fragilis* are known to secrete a formidable array of highly pro-inflammatory glycolipids, lipopolysaccharides (LPSs), lipooligosaccharides (LOS; smaller versions of LPS), barrier-disrupting enterotoxins such as BFT (*fragilysin)*, bacterial amyloids and small non-coding RNAs (sncRNAs) that are known to affect the structure and function of biophysiological barriers such as the gastrointestinal (GI) tract barrier and blood-brain barrier (BBB) [16–21,43–49,94,100]. It is important to point out that *B. fragilis* (and its complex repertoire of neurotoxins) is just one of the many hundreds of thousands of bacterial subtypes resident in the GI-tract microbiome, and under normal physiological conditions there might be expected to be potentially generated an exceedingly variable and noxious mixture of multiple bacterial neurotoxins from many different microbial species. Dietary fiber intake, environment and lifestyle represent highly integrated components contributing to the maintenance of the microbiome and the potential for a life-long and continuous supply of neurotoxins (dashed black lines); the potential for contribution of neurotoxins via feedback mechanisms to microbiome abundance, complexity and speciation and lifestyle are currently suspected but are not well understood (dashed black lines with question marks)

Environment and lifestyle are two highly integrated elements that help define the biological, biochemical, biophysiological and microbial niche in which each organism occupies. Our environment for example directly impacts human lifestyle choices which can have a critical, vital and determining influence on the health, complexity and dynamics of the GI-tract microbiome, and the microbial-derived supply of elements ranging from beneficial to detrimental and associated with homeostatic dysfunction and disease (Figure 1). These elements broadly impact glucose and energy metabolism, cellular, nuclear and metabolic function and homeostasis, the health of the GI-tract and BBB membranes and other biophysiological barriers, immunological, neuroendocrine and digestive activities and neurobehavioral development, maintenance and neurological function [80–82]. One continuing paradox of Westernized societies is that as the leading historical causes, constraints and limitations to human life expectancy are diminished or eradicated by progressive and significant advances in medicine, diseases related to poor dietary choices (including low fiber and high fat diets), sedentary life-styles, excessive and prolonged exposure to environmental toxins from toxic contaminants in our diet to antibiotics, food additives and food processing, health economics and age-related disease have now become major contributors to human mortality. The dynamics of the human GI-tract microbiome is becoming increasingly recognized as perhaps the most important contributor to many of these diseases especially through the provision of nutrients, the modulation of both active- and innate-immunity and the myriad of signaling intermediates that regulate diverse biological systems driven by the transcription, translation and the massive interaction and integration of both the human host and the microbial *‘hologenome’* acting together.

Very recently, in studies involving the functional and phylogenetic diversity associated with global populations, an extensive biostatistical- and bioinformatics-based analyses of the GI-tract *‘metagenomes’* of ~2100 human donors detected about ~22.3 million non-redundant prokaryotic genes, and *at least half of all of the genes identified were unique to the individual from which that GI-tract microbiome was derived* [9]. When compared to the established human genome content of 26.6 thousand protein-encoding transcripts of the human genome sequencing project obtained about ~18 years ago, the number of microbial genes in the human GI-tract microbiome alone was found to outnumber human genes by about 837 to 1 [9,83–85]. In a related study from a total of 9,428 global, body-wide human metagenomes, 154,723 microbial genomes and 2.85 million genes were annotated, and thousands of microbial genomes were identified from yet-to-be-named species [3,83]. Perhaps the most fascinating and novel findings were: **(i)** that ~50% of all of an individual’s microbial genetic make-up was found to be unique to that individual; and **(ii)** the huge genetic variation in many intestinal bacteria, including the human GI-tract abundant microbe *B. fragilis*. Overall this suggests that even for common, well-studied microorganisms a surprising amount of intra-species genomic diversity and associated biochemical functions still remains to be categorized. These findings continue to support the GI-tract microbiome as being an extremely active, dynamic and changing ecosystem dependent on the host’s age, diet, habits, environment, ethnicity, and health and/or disease status [86–89], and that lifestyle choices and environmental variables including for example, the host’s choice of geographical and environmental location has a significant bearing on both the dynamic composition of their microbiome and through this their overall health status [90–100].

## Use of probiotic and/or prebiotic to approaches to optimize human health

As our characterization and understanding of the human microbiome advances, there is emerging the intriguing possibility that the constitution of the GI-tract microbiome could be transiently or permanently altered through diet, dietary fiber intake, probiotics and/or prebiotics to optimize human health and both lower the incidence or even treat disease. This approach might be especially useful in those disorders resistant to pharmacological- or immunological-based therapies. For example, experiments in transgenic murine models as well as emerging human clinical studies have revealed that therapeutic manipulation of the microbiota, using fecal microbial transplantation, natural or engineered probiotics, or pre-biotics represent effective nontoxic and non-invasive approaches for the treatment, clinical management and/or prevention of for example, allergies, autoimmune diseases, *Clostridium difficile* infection and enhance the efficacy of certain cancer immune-therapeutics, especially in the elderly [21,30,89,95–100]. Preliminary results from our own laboratory using the 5xFAD amyloid-overexpressing murine mouse model of AD (containing 5 familial Alzheimer’s disease genes and based on a C57BL6 murine background) shows that animals fed high-fiber (both soluble and insoluble) diets have a lower abundance of *B. fragilis* in their GI-tract microbiome compared to age-matched control mice receiving standard diets, and show significant improvement in cognition and memory tasks as they age (manuscript in preparation).

## Summary

Over the last decade, our understanding of the immense contribution of GI-tract microbiome to human physiology and host metabolic functions has increased dramatically, yet progress is limited by the sheer complexity and dynamics of these microbial communities [3,81]. Microorganisms of the human GI-tract microbiome are now generally appreciated as playing some critical role in the maintenance of health and the development of disease, however the complexity and diversity of this ‘*dispersed organ system*’, and commensal and symbiotic relationships with human host cells, particularly with host cells of the central nervous system (CNS) remains incompletely understood. Neurotoxins detrimental to the normal structure, function and signaling properties of brain cells: **(i)** may be acquired directly via naturally-occurring, plant growth- and plant yield-promoting factors or processed components of ingested foodstuffs; **(ii)** from the environment; **(iii)** from the individual lifestyles that we live; and also **(iv)** from the neurotoxic exudates derived from thousands of species of stressed GI-tract resident microbes. Diet, environment and lifestyle are inextricably linked when considering ‘*hologenome*’ aspects of a highly networked ‘*metaorganism*’ and metagenomics factors with the most recent findings that microbiome-derived neurotoxins can strongly contribute to human diseases from intestinal and systemic inflammation, to obesity to schizophrenia and to Alzheimer’s disease (AD) [21,27–33,37–40]. For example, in fiber-deprived diets which can be strongly impacted by environmental and lifestyle choices certain GI-tract abundant Gram negative bacilli such as *B. fragilis* appear to strongly proliferate, increasing both their potential and abundance for the synthesis and release of neurotoxins by mass action alone. Importantly, especially against a background of unwise and unhealthy environmental and lifestyle choices, some of the most potent neurotoxins known can be a significant and a continuous *‘life-long’* source from the diet, in part through an insufficient supply of dietary fiber and the maintenance, support and proliferation of dysbiotic microbes that maintain their persistent and continual residence in the human GI-tract microbiome.
